# Book Reviews

**Published:** 1900-03

**Authors:** 


### BOOK REVIEWS.

### Proceedings A. V. M. A., 1899.

In shorter time than the period designated the Publication Committee, under the chairmanship of Dr. W. L. Williams, of Ithaca, New York, brought forth the proceedings of the annual convention in New York.

The rapid production of the same has not allowed it to be the perfection of the printer's art that adds so much of value to scientific works, nor has the haste of the editors made it as complete as it should have been.

Some papers of much importance, specially prepared for the New York meeting, are omitted entirely, others are severely abstracted, while the discussions as reproduced fail very much in many instances of being faithful reproductions of what was said and discussed.

It is very gratifying and pleasing, indeed, to have such matters promptly attended to, but it will not do to sacrifice completeness, accuracy, and perfection of production in a headlong rush for rapid reproduction. Scientific records of value are not made in this way.

### The Fourth Revised Edition of Gould.

Thirty thousand medical words, pronounced and defined, published by P. Blakiston's Son & Co., Philadelphia, at $1 per copy is one of the most valuable small medical dictionaries in print. Containing all the more important words, many that are not in daily use, with a series of valuable tables that make it of much service to the busy veterinarian, as a book of reference containing some 837 pages of solid matter, makes it the cheapest book of its kind published.

DISEASES OF POULTRY. By D. E. SALMON, D.V.M. Published by George E. Howard & Co., Washington, D. C.

THIS work, containing some two hundred and fifty pages, covers in a very careful and scientific manner the many diseases to which fowls are subject.

So much has been written on this subject by laymen and breeders, often the veriest trash, the most ridiculous conclusions arrived at, and bosh so far as scientific medical treatment is concerned, that it is refreshing to find one author whose contribution bears evidence of careful, intelligent study and whose conclusions are based upon scientific observations.

This great industry of poultry raising, representing in America alone an investment of upward of $350,000,000, is surely of sufficient importance to our people to have it considered and studied in a thoroughly scientific manner. The annual losses are extremely onerous and are in a great measure controllable, and in some instances should be wholly wiped out. Such works as Dr. Salmon's will aid very much in the preservation and extension of this widely distributed industry.

